# After-School to Pre-Sleep Energy Restriction for obesity in primary school children in grades 3–6: protocol for a parallel, randomized controlled trial

**DOI:** 10.1186/s13063-026-09785-2

**Published:** 2026-05-22

**Authors:** Xuemei Xie, Ying Lv, Xiyi Li, Yu He, Li Li, Xiuping Xuan, Xian Liu, Haiyan Yang, Xinghuan Liang

**Affiliations:** 1 Department of Endocrinology, The First Affiliated Hospital of Guangxi Medical University, Nanning, Guangxi 530021 China; 2 School of Public Health, Guangxi Medical University, Nanning, Guangxi China; 3 Department of Clinical Nutrition, The First Affiliated Hospital of Guangxi Medical University, Nanning, Guangxi China

**Keywords:** Pediatric obesity, Eating behavior, Energy intake, Randomized controlled trial

## Abstract

**Background:**

The rising prevalence of childhood obesity has become a major global public health concern. Dietary intervention remains the cornerstone of pediatric obesity management. However, both conventional all-day energy-restricted diets and time-restricted eating have limitations in school-aged children. Studies indicate that excessive evening caloric intake is closely associated with obesity. This trial aims to evaluate the efficacy of an After-School to Pre-Sleep Energy Restriction (ASER) strategy compared with standard dietary intervention for pediatric obesity management.

**Methods and analysis:**

This study is a prospective, randomized, open-label, parallel-group trial. We plan to enroll 164 obese children from grades 3 to 6, who will be stratified by age, gender, and BMI *z*-score and randomly assigned in a 1:1 ratio to either the ASER group or the control group. The 24-week intervention consists of an intensive phase (weeks 0–12) followed by a maintenance phase (weeks 13–24). The primary endpoint is the between-group difference in BMI *z*-score (BMIz) change at week 12. Secondary endpoints include within-group BMIz changes at week 12; between-group and within-group BMIz changes at week 24; between-group and within-group changes in %BMI_95_, body composition, growth parameters, cardiometabolic markers, and psychobehavioral metrics at both 12 and 24 weeks; and between-group differences in the incidence of adverse events at weeks 12 and 24.

**Ethics and dissemination:**

This study was approved by the Medical Ethics Committee of the First Affiliated Hospital of Guangxi Medical University (Approval No.: 2025-K0254). Written informed consent will be obtained from legal guardians, and assent will be obtained from child participants prior to enrollment. Study findings will be published in peer-reviewed journals.

**Trial registration:**

The trial was prospectively registered on 3 September 2025, prior to the first participant enrollment, with the Chinese Clinical Trial Registry (ChiCTR2500108702). The URL of the specific trial registry record is https://www.chictr.org.cn/showproj.html?proj=275435.

## Introduction

The global epidemic of childhood and adolescent obesity continues to escalate. Data from the Non-Communicable Diseases Risk Factor Collaboration (NCD-RisC) reveal a substantial increase in the global age-standardized obesity prevalence among school-aged children and adolescents (5–19 years) from 1990 to 2022, with rates rising from 1.7% to 6.9% in girls and from 2.1% to 9.3% in boys [1]. This public health crisis has extended beyond concerns of physical appearance, contributing to metabolic comorbidities and increased incidences of depression and anxiety [2–4]. Notably, obesity phenotypes established before puberty may induce metabolic memory effects through epigenetic modifications, significantly elevating the risk of cardiovascular diseases in adulthood [5, 6]. Therefore, timely and effective interventions targeting childhood obesity are critical for improving long-term outcomes.

Regarding strategies for obesity management, although bariatric surgery and novel pharmacotherapies such as GLP-1 receptor agonists have demonstrated remarkable efficacy in adults [7–10], their application in pediatric populations remains limited, largely due to insufficient long-term safety data concerning growth trajectories (e.g., bone mineral density), neurocognitive development, and psychosocial adaptation, as well as disparities in healthcare accessibility arising from unequal regional medical resources and socioeconomic factors [11–13]. Consequently, early lifestyle interventions, particularly dietary modifications, remain the cornerstone of clinical practice guidelines for managing childhood obesity across different countries.

Energy-restriction regimens, including continuous energy restriction (CER), intermittent energy restriction (IER), and very low energy diets (VLEDs), have been shown to induce weight loss by reducing overall caloric intake [14–17]. However, there is insufficient evidence regarding the long-term safety of stringent daily energy restrictions—especially VLEDs—in pediatric populations [18]. In addition, practical barriers—such as the inability of school meal programs to accommodate individualized low-energy dietary needs, the limited self-regulatory capacity of children, and the lack of consistent parental supervision in dual-working families—make adherence to all-day energy restriction particularly challenging [19, 20].

On the other hand, time-restricted eating (TRE), an emerging chrono-nutrition strategy, has demonstrated distinct advantages in weight management and metabolic regulation [21]. Nonetheless, current evidence for TRE is primarily derived from adult populations, and its strict feeding windows (e.g., requiring cessation of intake by 16:00) are often incompatible with the daily schedules of school-aged children. Crucially, high-quality evidence regarding the long-term safety of stringent TRE protocols on children’s growth, development and psychosocial outcomes remains lacking [22]. Thus, both conventional all-day energy restriction and TRE approaches exhibit limitations in pediatric applications. Contemporary interventions should be developmentally appropriate and aligned with children's daily routines to facilitate sustainable behavior change. Interventions that minimize implementation barriers while accommodating educational schedules and family dynamics are urgently needed for pediatric obesity management in real-world settings.

Accumulating evidence suggests that excessive nighttime caloric intake is closely associated with obesity in both adults and children [23–32]. For example, Longo-Silva et al. observed that individuals consuming their largest meal at dinner had higher BMI and increased risk of obesity [23]. A systematic review and meta-analysis revealed that pre-sleep high-energy intake was associated with increased obesity risk in children and adolescents [29]. Moreover, Garaulet et al. reported diminished weight loss efficacy in obese individuals consuming most daily calories after 15:00 [31]. Mechanistically, nighttime eating may exacerbate metabolic risks through enhanced fat storage, suppressed lipid oxidation, elevated LDL and total cholesterol levels, amplified hunger signaling, and reduced energy expenditure [33, 34]. These findings suggest the evening-to-sleep period as a critical window for metabolic intervention. Interventions targeting nighttime energy surplus may represent a promising and feasible strategy for weight management in obese children. To date, however, we have not identified any randomized controlled trials specifically investigating nighttime caloric restriction, rather than complete fasting, in obese pediatric populations.

In summary, considering children’s growth trajectories, dietary patterns, and prior research evidence, this randomized controlled trial evaluates an intervention model, after-school to pre-sleep energy restriction (After-school Energy Restriction, ASER), in obese children in grades 3–6. ASER focuses on limiting energy intake during the critical window from after school (approximately 17:30) to bedtime, permitting controlled energy intake tailored to age-specific nutritional requirements, coinciding with the period of peak caloric surplus. Rather than enforcing absolute fasting, ASER requires moderated energy intake after school, thereby aligning better with local cultural practices and daily living habits, while minimizing the risk of undernutrition and other adverse effects. This approach may reduce the burden of familial supervision, enhance children’s adherence to the intervention, and ultimately optimize its effectiveness.

## Methods and analyses

### Study design

This study is a prospective, randomized, open-label, parallel-controlled trial to be conducted at the First Affiliated Hospital of Guangxi Medical University. A total of 164 eligible participants will be recruited from non-boarding public primary schools in urban areas of Nanning and randomized in a 1:1 ratio into either the ASER group or the standard dietary intervention group (control group).

Participants will first undergo an initial screening based on predefined inclusion and exclusion criteria, including assessments of medical history, current medication use, and anthropometric measurements. Written informed consent will be obtained from the participants’ guardians before any study-specific procedures are performed.

Following consent, baseline evaluations will be conducted, including the collection of sociodemographic information, lifestyle factors, results from an oral glucose tolerance test (OGTT), insulin release test, lipid profile, and body composition analysis. Eligibility will be reconfirmed based on evaluation results, after which, participants will be randomized.

The intervention will last for 24 weeks, consisting of an initial 12-week intensive intervention phase followed by a 12-week maintenance phase. Participants assigned to the ASER group will be required to strictly restrict energy intake during the evening-to-bedtime period (starting at 17:30), while having no specific restrictions during other periods, although adherence to healthy eating patterns will be encouraged. Participants in the control group will receive standard weight management counseling in accordance with the Nutritional Guidelines for Weight Management in School-Aged Children [35].

**Fig. 1:**
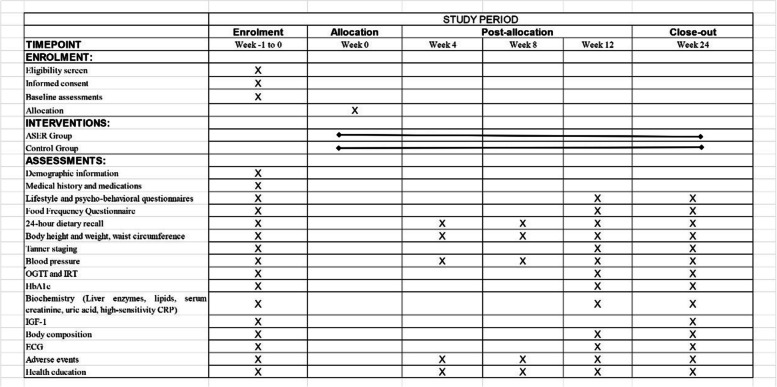
SPIRIT schedule of enrollment, interventions, and assessments. “X” denotes the timepoints at which each data collection activity is performed. ASER, After-School to Pre-Sleep Energy Restriction (After-school Energy Restriction); OGTT, oral glucose tolerance test; IRT, insulin release test; HbA1c, glycated hemoglobin; CRP, C-reactive protein; IGF-1, insulin-like growth factor 1; ECG, electrocardiogram

Throughout the study, both groups will receive additional lifestyle counseling beyond the dietary intervention, including recommendations on physical activity and sleep hygiene. The protocol has been developed following the SPIRIT 2013 statement (Standard Protocol Items: Recommendations for Interventional Trials) [36] (Fig. 1).

### Sample size estimation

Assumptions were informed by prior pediatric studies [14, 37], together with clinical considerations. We anticipate a between-group difference of 0.14 in BMI *z*-score change (standard deviation = 0.28) after 12 weeks of intervention. Assuming a two-sided significance level of 0.05, a statistical power of 85% (1–β = 0.85), and an expected dropout rate of 20%, a total of 164 participants (82 per group) will be required.

### Recruitment and informed consent

Participants will be recruited from eligible children with obesity attending non-boarding public primary schools in urban areas of Nanning. Recruitment is expected to take place from October 2025 through April 2026. All participants and their legal guardians will receive detailed information about the study procedures, potential benefits, and risks. Written informed consent will be obtained from legal guardians. In addition, assent will be obtained from children aged 8 years and older who demonstrate adequate comprehension. This study was approved by the Medical Ethics Committee of the First Affiliated Hospital of Guangxi Medical University (Approval No.: 2025-K0254).

#### Inclusion criteria

Participants meeting all of the following criteria will be included in the study:

1. Children aged 8 to 11 years, enrolled in grades 3 through 6 of primary school, at the time of providing informed consent
2. Diagnosed with obesity according to the diagnostic criteria outlined in the Chinese Health Industry Standard (WS/T586-2018) “Screening for Overweight and Obesity in School-age Children and Adolescents” [38].
3. Demonstrated clear willingness from both child and parent/guardian to participate in weight management interventions.
4. Written informed consent from the legal guardians and assent from the child participants obtained prior to enrollment.

#### Exclusion criteria

Participants meeting any of the following conditions will be excluded:

1. Cognitive impairment or psychiatric disorders such as depression.
2.  > 5 kg weight fluctuation within 3 months prior to enrollment
3. Currently enrolled in another weight-loss program or have taken medications that affect metabolism or body weight (such as glucocorticoids or antibiotics) in the past 3 months.
4. Diagnosed with any of the following conditions: diabetes; impaired liver function or active liver disease; impaired renal function or chronic kidney disease; active malignancy or history of malignancy within the past 5 years; malabsorption, functional gastrointestinal disorders; uncontrolled hyponatremia; hyperthyroidism; hypothyroidism; Cushing’s syndrome; eating disorders such as anorexia nervosa or bulimia nervosa; or any other diseases affecting body weight.
  o Definition of impaired liver function: alanine aminotransferase (ALT) and/or aspartate aminotransferase (AST) levels > 3 times the upper limit of the age- and sex-specific reference range [39].
  o Definition of impaired renal function: serum creatinine levels > the upper limit of the age- and sex-specific normal range [39].
5. History of food allergies or bariatric surgery; serious gastrointestinal disorders or gastrointestinal surgery within 12 months prior to randomization; smoking; known or suspected alcohol or recreational drug abuse; HIV infection; immunodeficiency; or any other condition that makes the participant unsuitable for the study.
6. Any other condition deemed inappropriate by investigators

#### Withdrawal or discontinuation criteria

Participant involvement in the study will be terminated under any of the following conditions:

1. Emergence of serious adverse events (related or unrelated to intervention) including diseases requiring excluded medications (e.g., systemic glucocorticoids), serious infectious diseases and any life-threatening conditions
2. Participant or legal guardian requests to withdraw consent, or the participant is unwilling to comply with the intervention protocol, or other unforeseen reasons.

### Randomization and allocation

Eligible participants will be randomized 1:1 to the ASER or control group using centralized, computer-assisted minimization with allocation concealment, balancing age (8–9 years vs. 10–11 years), sex (male vs. female), and baseline BMI *z*-score category (mild: + 1.645 ≤ z < + 2.5; moderate: + 2.5 ≤ z < + 3.0; severe: z ≥ + 3.0). To avoid treatment contamination, related participants from the same family will be allocated to the same intervention group. Randomization will be implemented by an independent statistician via a secure system; site investigators will enroll participants, submit baseline stratification variables, and receive the assignment, with the allocation sequence concealed from site staff until assignment. Due to the nature of the intervention, blinding of participants and intervention implementers is not feasible. However, health education physicians, data collectors, and outcome assessors/statisticians will remain blinded to group assignments throughout the study.

### Intervention measures

Children in the ASER group are required to consume no more than 30–35% of their target total daily energy intake during the period from after-school (17:30) until bedtime. The target total energy intake during the weight loss phase is based on the age-appropriate estimated energy requirement for healthy children, reduced by 300–500 kcal. Dinner must begin no later than 19:00 local time. Energy intake is not restricted during other time periods, but healthy eating guidance is provided, including recommended portions of fruits, vegetables, and dairy products to ensure adequate nutrition.

Dietitians will calculate dinner and post-dinner snack portions based on each child’s eating habits and the Acceptable Macronutrient Distribution Ranges (AMDR) recommended for children [40], and will develop individualized dietary plans. Nutrition education will be provided to the caregivers using food models or photographs. Caregivers are advised to weigh foods during meal preparation and to keep detailed records. To enhance compliance, one evening per week may be designated as a “free day,” during which energy intake is not strictly restricted.

The first 12 weeks constitute the intensive intervention phase, followed by a maintenance phase from weeks 13 to 24. In the first week, caregivers are required to record and submit daily dietary logs and food photographs via a designated WeChat group, with particular emphasis on the period from after-school to bedtime, to facilitate monitoring and guidance by the research team.

During the intensive intervention phase, dietary intake will be assessed at weeks 4 and 8 using 24-h dietary recalls. At these interim visits, participants will also receive face-to-face guidance, health education, and anthropometric measurements. In the maintenance phase, participants are expected to continue following the prescribed dietary plan.

Participants in the control group will receive standard clinical dietary advice for weight management based on the Nutritional Guidelines for Weight Management of School-Aged Children [35], which include recommendations on energy intake and healthy dietary patterns. The mode and frequency of feedback and supervision will be consistent with those of the intervention group. Both groups will receive regular health education on lifestyle topics, including balanced nutrition, physical activity, and sleep hygiene. Comprehensive assessments, including food frequency questionnaires (FFQ), 24-h dietary recalls, anthropometric measurements, body composition analysis, laboratory tests, and relevant questionnaires, will be conducted at baseline (week 0), week 12, and week 24. During the study period, participants will be asked not to engage in any additional weight-loss interventions, including other dietary programs, medications, or structured exercise regimens outside of their usual activities. Routine pediatric care and non-weight-related treatments will be allowed.

### Outcomes

All outcome data will be collected and analyzed by certified evaluators who are blinded to treatment allocation.

#### Primary outcome

The primary outcome is the between-group difference in body mass index *z*-score (BMIz) change at week 12 (ASER group vs. control group).

#### Secondary outcomes

1. Body weight and body composition indicators:
  BMIz: Between-group differences at week 24; within-group changes from baseline at weeks 12 and 24.
  Degree of reduction in %BMI_95_ (≥ 5%, ≥ 10%, and ≥ 15%), waist-to-height ratio, and body composition parameters (fat mass index [FMI], fat-free mass index [FFMI], visceral fat area, body fat percentage): between-group and within-group changes at weeks 12 and 24.
2. Growth and developmental indicators:
  Height, Tanner stage, annual growth velocity (GV), and height SDS changes from baseline, with between-group comparisons at weeks 12 and 24.
    GV (cm/year) = 12 * (current height – previous height)/(visit interval in months).
    Height SDS is calculated based on data from the 2005 Chinese National Growth Survey in nine cities [41]: Height SDS = (height at assessment time − mean height for same age/gender group)/standard deviation of height for same age/gender group).
  IGF-1 changes from baseline and between-group comparisons at week 24.
3. Cardiometabolic risk factors and serum biochemical indicators:
  Blood pressure; serum biochemical indicators (high-sensitivity C-reactive protein, liver function, renal function, uric acid); lipid profile (total triglycerides, HDL-C, LDL-C); blood glucose at 0, 60, and 120 min during the oral glucose tolerance test (OGTT); insulin release test (IRT); area under the curve (AUC) for OGTT and IRT; homeostasis model assessment of insulin resistance (HOMA-IR) and β-cell function (HOMA-B): between-group and within-group changes at weeks 12 and 24
4. Dietary and psychobehavioral parameters:
  Dietary quality (dietary balance index, dietary inflammatory index, etc.), dietary patterns, sleep, anxiety levels, and physical activity: between-group and within-group changes at weeks 12 and 24.
5. Adverse events (AEs):
  AEs will be systematically monitored and recorded throughout the trial. Expected AEs in this low-risk intervention are mild.
  Between-group differences in the incidence of all AEs at weeks 12 and 24.

### Questionnaire survey

Trained investigators who are blinded to group allocation will collect data using validated questionnaires to assess personal information (e.g., name, sex, age), medical history, intake of macronutrients and energy, daily physical activity, sleep patterns, and anxiety levels. Dietary quality and patterns will be evaluated using 24-h dietary recall, food frequency questionnaires (FFQ), and indices such as the dietary balance index (DBI) and the dietary inflammatory index (DII).

### Anthropometric and blood pressure measurements

Height and weight will be measured by trained assessors blinded to group allocation. Height will be measured to the nearest 0.1 cm using a calibrated wall-mounted stadiometer, and body weight will be measured to the nearest 0.1 kg using a calibrated digital scale at each study visit. Waist circumference will be measured at the midpoint between the lowest rib and iliac crest using a standard, non-stretchable measuring tape to the nearest 0.1 cm, with participants standing upright at normal expiration. All anthropometric measurements, including height, weight, and waist circumference, will be taken twice, and the average of the two readings will be used for analysis.

Blood pressure will be measured under standardized conditions by trained, blinded assessors using an automated oscillometric device (Omron, Japan). At least two readings will be taken at 1–2-min intervals, and the mean of the two readings will be used for analysis. If the difference between the first and second readings exceeds 5 mmHg, a third measurement should be taken. The average of all three readings should then be recorded as the final result.

### Laboratory assessments

All blood samples will be collected by trained and blinded nurses at the First Affiliated Hospital of Guangxi Medical University following standard operating procedures. Participants will be required to fast overnight (≥ 10 h) prior to sampling. All laboratory analyses will be performed at KingMed Diagnostics, a qualified third-party medical testing center, using the following validated methods described below. For the oral glucose tolerance test (OGTT) and insulin release test (IRT), fasting blood samples will be collected first. Within 5 min, participants will ingest 1.75 g of glucose per kilogram of body weight (not exceeding 75 g), dissolved in 250 mL of water. Subsequent blood samples will be drawn at 60 min and 120 min after glucose ingestion.

Plasma glucose concentrations will be measured using the hexokinase enzymatic method (Mindray, China). Glycated hemoglobin (HbA1c) will be measured by high-performance liquid chromatography (HbA1c Analyzer, Lifotronic Technology, China). Serum insulin levels will be assessed using a chemiluminescence immunoassay (Mindray, China). Insulin-like growth factor 1 (IGF-1) will be measured using liquid chromatography-tandem mass spectrometry (LC-MS/MS; Thermo Fisher Scientific, USA). High-sensitivity C-reactive protein (hs-CRP), liver enzymes, renal function markers, uric acid, and lipid profile will be analyzed using an automated biochemical analyzer (Mindray, China).

Additionally, with participant consent, certain specimens (including whole blood and serum) will be stored at − 80 °C for potential future studies, such as metabolomics or transcriptomic sequencing. These future studies have not yet been determined, and the use of stored samples will depend on specific research needs. This has been clearly outlined in the informed consent form. Any future use of the samples for related analyses will require separate approval from the ethics committee, and results from exploratory analyses will be reported independently from the main study findings.

### Body composition and additional assessments

Body composition, including fat mass index (FMI), fat-free mass index (FFMI), visceral fat area, and body fat percentage, will be measured using a bioelectrical impedance analyzer (InBody, South Korea). Electrocardiography (ECG) will be performed to assess for potential arrhythmias or conduction abnormalities.

### Adverse events and safety monitoring

No formal interim analyses or stopping guidelines are planned; all data will be analyzed post-follow-up. However, the principal investigator (PI) will suspend the trial immediately if ≥ 3 intervention-related serious AEs (SAEs) occur in one group, with final termination requiring PI and IRB consensus. This ensures participant safety while aligning with predefined secondary endpoints for AE incidence.

An adverse event (AE) is defined as any unfavorable sign, symptom, or medical condition that arises after the initiation of the study intervention. Pre-existing medical conditions or diseases will only be considered AEs if they worsen after the start of the intervention. Unchanged pre-existing conditions will not be classified as AEs. Abnormal laboratory findings will not be considered AEs unless deemed clinically significant by the investigator.

At each clinical visit, the investigator (or a designated staff member) will assess whether any AEs have occurred. If a participant is unable to continue in the study due to an AE or other unavoidable reason, the intervention will be discontinued. Reasons for discontinuation may include gastrointestinal symptoms or growth and developmental issues that, in the opinion of the physician, warrant termination of the intervention.

All AEs will be evaluated and managed by the participant’s physician and monitored until resolution to ensure participant safety. AEs will be systematically documented according to standardized procedures. The incidence of AEs is predefined as a secondary endpoint to support a comprehensive benefit-risk assessment of the intervention.

### Quality control and data management

Data will be collected using paper Case Record Forms (CRFs). Prior to participant enrollment, all study personnel will receive standardized training to ensure proper understanding of study procedures and data recording requirements. Data will be recorded by trained investigators in real time, followed by independent double-checking by two individuals to ensure completeness and logical consistency of the forms. Data entry will be performed using a double-entry system. Upon completion, consistency checks will be conducted. Any discrepancies or abnormal values will be traced back to the original records for verification and correction. All participants will be identified using unique study codes. The linkage between personal information and study codes will be stored separately, accessible only to authorized research staff for necessary identification. All paper-based data will be securely stored in designated cabinets, with no access granted to unauthorized personnel. The hospital’s Ethics Committee will oversee the data collection and management process to ensure compliance with ethical guidelines and data protection requirements.

To promote participant retention and adherence to the follow-up schedule, regular reminders (e.g., phone calls or text messages) will be provided before each study visit. Participants and their guardians will receive educational support and periodic feedback regarding progress. Concurrent health education sessions during visits will reinforce engagement and study goal understanding. For participants who withdraw or deviate from the intervention protocol, efforts will be made to collect primary and secondary outcome data at all available time points, particularly at the 12-week and 24-week assessments.

### Oversight and monitoring

Given the minimal risk nature of the intervention and the small, single-center design, a formal Data Monitoring Committee (DMC) was deemed unnecessary. No formal auditing is planned. Study oversight will be maintained by the principal investigator and co-investigators. The study will not have a separate coordinating center, steering committee, or endpoint adjudication committee. The hospital’s Medical Ethics Committee will provide ethical oversight, ensuring that the study is conducted in compliance with ethical guidelines. The Research Administration Department of the hospital will monitor the administrative and financial compliance of the study.

### Protocol amendments

Any important protocol amendments (e.g., to eligibility criteria, outcomes, or analysis methods) will be promptly communicated to all relevant parties, including investigators, the Medical Ethics Committee, trial registries, and participants as appropriate.

### Statistical analysis

Data analysis will follow the intention-to-treat (ITT) principle and will be conducted under blinded conditions. For the primary endpoint (between-group difference in BMI *z*-score change at week 12) and secondary endpoints involving continuous variables (BMI *z*-scores, waist-to-height ratios, body composition parameters, cardiometabolic markers, and psychobehavioral measures, etc.) at weeks 12 and 24, longitudinal changes will be assessed using mixed-effects models. These models will account for within-participant correlations and missing data. A time-by-group interaction term will be included to evaluate whether there are significant differences in weight reduction between the study groups. The models will be adjusted for stratification factors (age, sex, and baseline BMI *z*-score) and corresponding baseline values to control for any potential systematic group differences at baseline.

Continuous variables will be compared using independent-sample *t*-tests or Mann–Whitney *U* tests, as appropriate. Categorical outcomes, including the proportions achieving predefined reductions in %BMI95 (≥ 5%, ≥ 10%, ≥ 15%) and AE incidence, will be analyzed using chi-square tests and logistic regression. Sensitivity analyses will be conducted to test the robustness of the results. A post hoc analysis will compare adherers and non-adherers, with adherence defined as attendance at ≥ 50% of scheduled visits during the 24-week study period. Exploratory dietary adherence will be defined at the participant level as not exceeding the group-specific upper limit at ≥ 50% of available post-baseline recalls. Participants with fewer than two post-baseline recalls will be considered not classifiable for dietary adherence analyses. Statistical significance will be set at a two-tailed *P*-value of < 0.05. All analyses will be performed using SPSS and R software.

## Discussion

Childhood obesity is a growing global public health concern, linked to a wide range of metabolic health risks [2–6]. Although traditional all-day caloric restriction interventions, such as CER, remain a cornerstone of obesity management, concerns persist regarding their long-term safety and adherence in pediatric populations [18–20]. While studies have investigated all-day caloric restriction or complete fasting windows in pediatric populations, to the best of our knowledge, no published RCT has specifically examined the efficacy of a targeted partial energy restriction during the after-school to pre-sleep period, while still permitting controlled intake to maintain feasibility in school-aged children. The approach proposed in this protocol, After-School to Pre-Sleep Energy Restriction (After-school Energy Restriction, ASER), targets this critical window of excess consumption. By moderating rather than eliminating intake during this period, we aim to address gaps in existing approaches while aligning with children’s daily routines and cultural practices. We hypothesize ASER will improve BMI *z*-scores and other obesity-related health markers, with its flexible design potentially enhancing adherence and intervention efficacy.

This study has several strengths. First, it leverages emerging evidence linking nighttime caloric surplus to metabolic dysregulation [23–32] but avoids the stringency of traditional all-day caloric restriction and TRE protocols (e.g., early feeding cutoffs), which are hard to implement for school-aged children. Previous research has shown that diet interventions for childhood obesity often fail due to a lack of sustained engagement, with common causes being scheduling conflicts and programs failing to meet family needs, such as lack of flexible timing arrangements or failure to consider families’ financial circumstances [42]. By limiting the time window for caloric intake and incorporating weekly “free day,” we aim to make the intervention more manageable and engaging for children and their families. Second, by permitting controlled energy intake (30–35% of daily targets) during the evening period, the intervention balances practical feasibility with metabolic efficacy. This approach may mitigate common barriers to adherence, such as hunger-driven noncompliance in strict TRE regimens or the impracticality of all-day caloric restriction in school settings. Mechanistically, ASER aligns with circadian biology: evening-restricted feeding may enhance lipid oxidation and reduce nocturnal insulin resistance, potentially disrupting the “metabolic memory” associated with childhood obesity [33, 34]. Third, a comprehensive set of outcomes—including anthropometric measures, cardiometabolic risk factors, psychobehavioral and dietary parameters by blinded evaluators, allowing holistic evaluation of intervention effects beyond simple weight metrics. Moreover, the study’s reliance on a comprehensive support system, including parental involvement, lifestyle counseling, and personalized dietary plans, may provide a robust framework to ensure both short-term and long-term success. Additionally, the caloric restriction threshold and macronutrient adjustments based on AMDR guidelines minimize undernutrition risks. Safety monitoring for growth trajectories and psychological well-being further ensures ethical rigor.

Despite its strengths, this study has several limitations. The open-label design and the lack of blinding of participants may introduce bias in terms of self-reporting behaviors and outcomes. Additionally, while the study includes a comprehensive set of outcome measures, the long-term sustainability of weight loss and health improvements remains uncertain, as the intervention lasts only 24 weeks. Further research with longer follow-up periods is necessary to confirm the durability of the results.

In conclusion, this protocol presents a promising approach to managing childhood obesity through an evening caloric control strategy. If successful, ASER could offer a viable and less restrictive alternative to existing dietary interventions, with the potential to improve the physical and psychological well-being of children affected by obesity. The results of this trial may inform future pediatric obesity treatment guidelines and contribute to the growing body of evidence supporting tailored, sustainable dietary interventions for children.

### Ancillary and post-trial care

No specific ancillary or post-trial care is planned beyond the provision of final health reports to participants. As a low-risk behavioral intervention, serious harm is not anticipated. Should any trial-related injury occur, compensation would be handled through the established medical liability system in accordance with Chinese regulations.

## Trial status

The study protocol (Version 20.3, dated November 17, 2025) is being used. As of the date of this submission, the study is in the pre-recruitment (preparatory) phase. Participant recruitment is expected to commence in late December 2025 or early January 2026.

Version note:

Version 20.0 (June 16, 2025): This was the version approved by the ethics committee.

Version 20.1 (June 25, 2025): This version reflects minor editorial refinements made in response to an external peer review organized by our funding agency, the Beijing Health Alliance Charitable Foundation. The scientific integrity of the protocol was maintained, with no modifications to the study objectives, design, eligibility criteria, interventions, methods of data collection, or statistical analysis plans.

Version 20.2 (October 20, 2025): This version included additional editorial adjustments made post-registration for the purpose of publication.

Current Submission - Version 20.3 (November 17, 2025): This version adds Keywords, a description of the trial status and version notes, to meet the requirements of this journal.

None of these revisions involved any modifications to the study objectives, design, eligibility criteria, interventions, data collection methods, or statistical plans.

## Data Availability

No datasets were generated or analysed during the current study.

## Abbreviations

ASER: After-School to Pre-Sleep Energy Restriction (After-school Energy Restriction)
BMI: Body mass index
BMIz: Body mass index *z*-score
CER: Continuous energy restriction
VLEDs: Very low energy diets
TRE: Time-restricted eating
OGTT: Oral glucose tolerance test
IRT: Insulin release test
HbA1c: Glycated hemoglobin
HDL-C: High-density lipoprotein cholesterol
LDL-C: Low-density lipoprotein cholesterol
IGF-1: Insulin-like growth factor 1
FMI: Fat mass index
FFMI: Fat-free mass index
AE: Adverse event
IRB: Institutional Review Board
PI: Principal investigator
AMDR: Acceptable Macronutrient Distribution Ranges
SPIRIT: Standard Protocol Items: Recommendations for Interventional Trials
